# Evaluation of Analgesic and Anti-Inflammatory Activity of Chloroform and Methanol Extracts of *Centella asiatica* Linn

**DOI:** 10.1155/2013/789613

**Published:** 2013-11-21

**Authors:** Sudipta Saha, Tanmoy Guria, Tanushree Singha, Tapan Kumar Maity

**Affiliations:** ^1^Department of Pharmaceutical Sciences, Babasaheb Bhimrao Ambedkar University, Vidya Vihar, Rai Bareli Road, Lucknow 226025, India; ^2^Department of Pharmaceutical Technology, Jadavpur University, Kolkata 700032, India

## Abstract

A variety of active constituents with wide range of pharmacological actions have been reported with *Centella asiatica*. The present study was undertaken to assess analgesic and anti-inflammatory properties of its leaf extracts. Dried leaves were defatted with petroleum ether and extracted with chloroform and methanol. Both chloroform and methanol extracts were evaluated for analgesic activity through tail clip, tail flick, tail immersion, and writhing assay tests at doses of 50, 100, and 200 mg/kg using Swiss albino mice. On the other hand, anti-inflammatory assay was performed by carrageenan induced paw edema of methanol extract at 100 and 200 mg doses in Wistar albino rat. Dextropropoxyphene and indomethacin were employed as a standard for analgesic and anti-inflammatory studies, respectively. Our present study demonstrated that *Centella asiatica* bears significant analgesic and anti-inflammatory activities in those models.

## 1. Introduction 

Human beings have relied on natural products as a resource of drugs for thousands of years. Plant-based drugs have formed the basis of traditional medicine systems that have been used for centuries in many countries such as Egypt, China, and India [1]. Today plant-based drugs continue to play an essential role in health care. It has been estimated by the World Health Organization that 80% of the population of the world rely mainly on traditional medicines for their primary health care [2]. Currently, at least 119 chemicals, derived from 90 plant species, can be considered as important drugs in one or more countries [3]. 

Nonsteroidal anti-inflammatory drugs (NSAIDs) are the most commonly used drugs to treat pain and inflammatory conditions [4]. But they have common side effects like ulcer, bleeding, and renal disorders [5]. Therefore, medicinal plants are the common source of therapeutically active chemical substances with lesser side effects [6].

*Centella asiatica* belongs to the family Apiaceae and is commonly found in parts of Asia and the Middle East. *Centella* has been used in traditional medicine in Asia for 100 years [7]. The major bioactive constituents are triterpene saponins mainly asiaticoside, sapogenin, asiatic acid, madecassoside, and madecassic acid [8]. It is believed to have beneficial effects in improving memory and treating mental fatigue, anxiety, and eczema [9]. In Ayurveda, *Centella* is effectively used in the treatment of inflammation, anaemia, asthma, blood disorders, bronchitis, fever, urinary discharge, and splenomegaly [10]. The aqueous extract of *Centella* possesses antioxidant, cognitive enhancing, and antiepileptic properties [11].

Based on the above findings, analgesic and anti-inflammatory activities were evaluated in this present study. These findings justified the traditional use of *Centella asiatica* in the treatment of inflammatory conditions. 

## 2. Methods

### 2.1. Plant Material and Preparation of the Extracts

The leaves were collected from costal area of West Bengal in the rainy season, authenticated by Botanical Survey of India, Howrah, India, and a voucher specimen number 124 was deposited for future references. The leaves were dried under shade for seven days and coarsely ground. 500 gm of powdered leaves were extracted with petroleum ether (60–80°C) for successive extraction. Then, the marc was dried and extracted with chloroform and methanol simultaneously. The extract was concentrated under vacuum and concentration was continued to completely remove the solvent. The percentage yields (% W/W) of petroleum ether, chloroform, and methanol extracts were 2.8, 3.8, and 13.8, respectively.

### 2.2. Phytochemical Screening

Various phytochemical tests were performed to determine the presence of alkaloids, steroids, triterpenoids, flavonoids, saponins, tannins, reducing sugars, and anthraquinones in extracts [12]. 

### 2.3. Acute Toxicity Studies

The acute oral toxicity studies of the extracts were performed as per revised Organisation for Economic Cooperation and Development (OECD) guidelines 423. Animal ethics committee approval was obtained for animal experiment (Registration number 0367/01/C/CPCSEA). The chloroform and methanol extracts of whole aerial parts/leaves of *Centella asiatica* was dissolved in water and administered orally to overnight fasted animals at the doses of 250, 500, and 1000 mg/kg of body weight in mice. After administration of the extracts, the animals were observed continuously for the first two hours, for any toxic manifestation. Thereafter, observations were made at regular intervals for 48 hours. Further, the animals were under investigation up to a period of 2 weeks for mortality and general behavior [13]. All observations were systematically recorded and maintained for each mouse individually.

### 2.4. Analgesic Activity of Chloroform and Methanol Extracts in Mice

#### 2.4.1. Experimental Animals

Swiss albino mice (18 to 20 gm) of either sex were obtained from the Rita enterprise, Kolkata. Animal ethics committee approval was obtained for animal experiment (Registration number 0367/01/C/CPCSEA). The animals were maintained under environmental condition and had free access to standard diet and fresh water ad libitum. They were housed in animal cages in an air condition area at 25 ± 2°C with 12 h light and dark conditions. The optimum conditions for experiments were decided on the basis of pilot experiments and carried out using six animals for each group. They were in fasting condition for 18 hours before starting the experiments. Dextropropoxyphene was used as positive control which collected from Cadila Pharmaceuticals, Ahmedabad, Gujrat.

#### 2.4.2. Mouse Writhing Assay

According to Koster et al. [14], the total number of writhes was counted after intraperitoneal injection of acetic acid (0.6% V/V in normal saline, 10 mL/kg) for 15 min, starting after 5 min of injection. The extracts were administered orally at 50, 100, and 200 mg/kg doses 30 min prior to acetic acid injection. Number of writhing and stretching was observed and recorded and expressed as percentage inhibition.

#### 2.4.3. Tail Flick Method

A modification of the method originally described by Pizziketti et al. [15] was employed. Mice were closely restrained in a wire mesh cage and the lower half of the tails dipped in cold water (4°C). The time (in seconds) for tail withdrawal from the water was taken as the reaction time. Measurement of threshold was made 30 and 60 min before and after administration of dextropropoxyphene (65 mg/kg, subcutaneously) or extracts (50, 100, and 200 mg/kg, per oral), respectively. Distilled water (10 mL/kg) served as the control.

#### 2.4.4. Tail Immersion Method

Mice were divided into groups of six animals each. The lower 5 cm portion of the tail was immersed in a beaker of water maintained at 55 ± 0.5°C [16]. The time in seconds for tail withdrawal from the water was taken as the reaction time, with a cut-off time of immersion at 10 sec. The reaction time was measured 1 hour before and after oral administration of extracts (50, 100, and 200 mg/kg, per oral) or distilled water (10 mL/kg). Dextropropoxyphene (65 mg/kg) was administered subcutaneously, 30 min before the test.

#### 2.4.5. Haffner's Tail Clip Method

A metal artery clip was applied to root of the mice tail to induce pain [17]. A sensitivity test was carried out and animals that were not attempted to dislodge the clip within 10 sec were discarded. The responsive mice were allotted to groups of six animals each. The tail clip was applied 60 min after oral administration of extracts (50, 100, and 200 mg/kg, per oral) or 30 min after subcutaneous administration of dextropropoxyphene (65 mg/kg). Distilled water (10 mL/kg) was served as the control.

### 2.5. Anti-Inflammatory Activity of Methanol Extract Using Carrageenan Induced Paw Edema in Rats

#### 2.5.1. Experimental Animals

Wistar albino rats (150 to 200 gm) of either sex were originally obtained from the Rita enterprise, Kolkata. The animals maintained under environmental conditions had free access to standard diet. They were housed in animal cages in an air conditioned area at 25 ± 2°C with 10/14 h light/dark cycle. The optimum conditions for experiments were decided on the basis of pilot experiments carried out using six animals for each group. They were in fasting condition for 18 hours before starting the experiments. Indomethacin was used as standard control which was obtained and collected from Cadila Pharmaceuticals, Ahmedabad, Gujrat.

#### 2.5.2. Procedure

The methanol extract of *Centella asiatica* leaves (100 and 200 mg/kg) or indomethacin (10 mg/kg) was administered orally to differentiate groups of rats as shown in Table 6. Acute inflammation was induced half an hour after treatment by subplanter injection of 0.1 mL freshly prepared 1% suspension of carrageenan in right hind paw of rats [18]. The paw volume was measured initially and then at 1, 2, 3, and 4 h after the carrageenan injection by using plethysmographic method [19].

### 2.6. Statistical Analysis

Statistical analysis was carried out using Graph Pad Prism 5.0 (Graph Pad Software, San Diego, CA). All results were expressed as mean ± SD. The data was analysed by one-way ANOVA followed by Bonferroni multiple comparison test. 

## 3. Results 

### 3.1. Acute Toxicity Studies

No mortality and behavioral changes were observed up to 2 weeks. Therefore, chloroform and methanol extracts were safe up to 1000 mg/kg body weight dose. In accordance with this test, *Centella asiatica* was tested at 50, 100, and 200 mg/kg body weight for further experiments. 

### 3.2. Phytochemical Screening

Phytochemical screening of both chloroform and methanol extracts showed that they contain saponins and tannins, whereas methanol extract also contained flavonoids and triterpenoids (Table 1). 

### 3.3. Analgesic Activity in Mice

#### 3.3.1. Acetic Acid-Induced Writhing Test

Both chloroform and methanol extracts exerted a dose-dependent decrease in abdominal constriction in all three doses in mice by using 1% acetic acid solution. The dose-dependent protective effects of chloroform extract exhibited ~1.5 times higher than methanol extract (Table 2). The protective effect for dextropropoxyphene was ~60% (standard drug) and this action was comparable with chloroform extract at 100 and 200 mg/kg doses.

#### 3.3.2. Tail Withdrawal Reflexes Induced by Tail Flick Test

According to Table 3, both extracts of *Centella asiatica *exerted maximum ~30% inhibition during tail flick method at 200 mg/kg doses, whereas dextropropoxyphene showed ~45% inhibition at therapeutic dose (65 mg/kg) in mice. The tail withdrawal reflexes were gradually increased from ~15 to ~30% during oral administration of extracts. 

#### 3.3.3. Tail Withdrawal Reflexes Induced by Tail Immersion Test

Tail immersion test is also another parameter for analgesic activity. All three doses of both extracts showed significant inhibition with respect to control. The positive control, dextropropoxyphene demonstrated ~50% inhibition at therapeutic dose, whereas both extracts showed ~25% inhibition up to 200 mg/kg dose. According to Table 4, it was evident that both extracts had significant analgesic activity which was slightly lower than dextropropoxyphene.

#### 3.3.4. Tail Withdrawal Reflexes Induced by Tail Clip Test

Tail clip test is another method for screening analgesic activity. According to Table 5, both extracts of *Centella asiatica* exerted maximum ~50% inhibition during tail clip method at 200 mg/kg doses, whereas dextropropoxyphene showed ~60% inhibition at therapeutic dose. It was observed that both extracts showed the highest inhibition in this method than any other models.

### 3.4. Anti-Inflammatory Activity of Methanol Extract Using Carrageenan Induced Rat Paw Edema in Rats

The paw volumes and percentages of inhibition by the chloroform and methanol extract of *Centella asiatica* and standard drugs are shown in Table 6. Carrageenan injection was administered one hour after treatment of extracts at three doses (50, 100, and 200 mg/kg) and indomethacin. Measurement of paw size was taken before carrageenan injection and then 1, 2, 3, and 4 h after carrageenan injection. It was observed that the methanolic extract showed significant inhibition at two doses. The inhibition was the highest at 3 h at 200 mg/kg dose which was slightly lower than indomethacin effect. 

## 4. Discussion

This study is the first report related to analgesic and antiinflammatory activity of *Centella asiatica* leave extracts. The analgesic activity was evaluated through acetic acid writhing, tail flick, tail clip, and tail immersion assays in mice, whereas antiinflammatory activity was performed through carrageenan induced paw edema in rats. We did not include the petroleum ether extract for our study because phytochemical screening revealed that there was no secondary metabolites in this extract. 

The antinociception activity was evaluated by acetic acid-induced writhing responses. The intraperitoneal administration of acetic acid produced both peripheral and central nociception action which acted through release of endogenous mediators and blocked by nonsteroidal anti-inflammatory drugs [20]. Both extracts showed significant reduction in writhing and stretching induced by acetic acid. Chloroform extract had higher inhibitory action at 200 mg/kg dose which is comparable to standard drug dextropropoxyphen (Table 2). Other doses of chloroform and methanol extracts also showed significant inhibition % writhing in mice. This action might be due to blockade of the release of endogenous substances. 

The brain and spinal cord play an important role in central pain mechanism. The dorsal part of the spinal cord is rich with substance P, endogenous opioids, somatostatine, and other inhibitory hormones which are the targets of pain and inflammation [21]. It is also established that tail clip, tail flick, and tail immersion models are the well-established methods for measuring the central analgesic effects of drugs through opoid receptor [21]. Our present study demonstrated that both chloroform and methanol extracts were effective against all these models at 200 mg/kg doses which were comparable with standard drug dextropropoxyphene (Tables 3–5). Narcotic analgesics are active against both peripheral and central pain, while nonsteroidal anti-inflammatory drugs inhibit peripheral pain [22]. Our findings suggested that both extracts may act like narcotic analgesic drugs. 

Methanol extract had significant anti-inflammatory effect against carrageenan-induced paw edema model, whereas chloroform extract had not such effect on rats. Carrageenan induced inflammation is a well-established method to detect orally active anti-inflammatory agents which shows biphasic response. The first phase is mediated through the release of histamine, serotonin, and kinins, whereas the second phase is through the release of prostaglandins [23]. Methanol extract at 200 mg/kg dose showed maximum inhibition at 2 h (Table 6); this finding signified that anti-inflammatory action might be due to inhibiting the release of histamine or kinins. 

## 5. Conclusion

The present study showed significant analgesic effect of both chloroform and methanol extracts at 100 and 200 mg/kg doses in mice, whereas methanol extract at 200 mg/kg dose had anti-inflammatory effect. We reported for the first time analgesic and anti-inflammatory effect of *Centella asiatica* in various models. 

## Figures and Tables

**Table 1:** Phytochemical screening of petroleum ether, chloroform and methanol extracts.

Test	Petroleum ether ext.	Chloroform ext.	Methanol ext.
Alkaloids	−	−	−
Steroids	−	−	−
Triterpenoids	−	−	+
Flavonoids	−	−	+
Saponins	−	+	+
Tannins	−	+	+
Reducing sugar	−	−	−
Amino acids	−	−	−
Anthraquinones	−	−	−

(+) and (–) signified present and absent, respectively.

**Table 2:** Effect of *Centella asiatica* extracts (chloroform and methanol) on acetic acid-induced writhing behavior in mice.

Drugs (dose)	Methanol		Chloroform	
	No. of writhing	% Inhibition	No. of writhing	% Inhibition
Control (saline)	49.60 ± 5.12	0.00	49.60 ± 5.12	0.00
Dextropropoxyphene (65 mg/kg)	17.40 ± 2.53^b^	64.91	17.40 ± 2.53^b^	64.91
Extract (50 mg/kg)	39.00 ± 1.04^a^	21.37	22.40 ± 2.67^a^	54.28
Extract (100 mg/kg)	37.60 ± 2.07^a^	24.19	18.20 ± 2.02^a^	62.85
Extract (200 mg/kg)	29.80 ± 0.77^b^	39.91	17.70 ± 2.92^b^	64.31

Values are mean ± SEM (*n* = 6). Differences in groups were analyzed by one-way analysis of variances (one-way ANOVA) followed by Bonferroni multiple comparison test. ^a^ *P* < 0.05 and ^b^ *P* < 0.001 represented highly significant compared to control.

**Table 3:** Protective effect of *Centella asiatica* extracts (methanol and chloroform) on tail withdrawal reflexes induced by tail flick method in mice.

Drugs (dose)	Methanol			Chloroform	
	Before treatment (sec)	After treatment (sec)	% Inhibition	After treatment (sec)	% Inhibition
Control (saline)	5.53 ± 0.15	5.53 ± 0.15	0.00	5.53 ± 0.15	0.00
Dextropropoxyphene (65 mg/kg)	5.58 ± 0.35	10.74 ± 0.52^b^	48.04	10.74 ± 0.52^b^	48.04
Extract (50 mg/kg)	5.57 ± 0.19	6.64 ± 0.19^a^	16.11	6.34 ± 0.56^a^	12.14
Extract (100 mg/kg)	6.06 ± 0.37	7.73 ± 0.45^a^	21.60	7.05 ± 0.44^a^	14.04
Extract (200 mg/kg)	6.07 ± 0.33	8.45 ± 0.54^a^	28.16	8.63 ± 0.40^a^	29.66

Values are mean ± SEM (*n* = 6). Differences in groups were analyzed by one-way analysis of variances (one-way ANOVA) followed by Bonferroni posttest. ^a^ *P* < 0.05 and ^b^ *P* < 0.001 represented highly significant compared to control.

**Table 4:** Protective effect of *Centella asiatica* extracts (methanol and chloroform) on tail withdrawal reflexes induced by tail immersion method in mice.

Drugs (dose)	Methanol			Chloroform	
	Before treatment (sec)	After treatment (sec)	% Inhibition	After treatment (sec)	% Inhibition
Control (saline)	5.53 ± 0.15	5.53 ± 0.15	0.00	5.53 ± 0.15	0.00
Dextropropoxyphene (65 mg/kg)	5.58 ± 0.35	10.74 ± 0.52^b^	48.04	10.74 ± 0.52^b^	48.04
Extract (50 mg/kg)	5.57 ± 0.19	6.64 ± 0.19^a^	19.21	6.19 ± 0.16^a^	10.01
Extract (100 mg/kg)	6.06 ± 0.37	7.73 ± 0.45^a^	21.60	6.96 ± 0.21^a^	12.93
Extract (200 mg/kg)	6.07 ± 0.33	8.45 ± 0.54^a^	28.16	7.91 ± 0.37^a^	23.26

Values are mean ± SEM (*n* = 6). Differences in groups were analyzed by one-way analysis of variances (one-way ANOVA) followed by Bonferroni posttest. ^a^ *P* < 0.05 and ^b^ *P* < 0.001 represented highly significant compared to control.

**Table 5:** Protective effect of *Centella asiatica* extracts (methanol and chloroform) on tail withdrawal reflexes induced by tail clip method in mice.

Drugs (dose)	Methanol			Chloroform	
	Before treatment (sec)	After treatment (sec)	% Inhibition	After treatment (sec)	% Inhibition
Control (saline)	1.27 ± 0.07	1.27 ± 0.07	0.00	1.27 ± 0.07	0.00
Dextropropoxyphene (65 mg/kg)	1.21 ± 0.06	3.17 ± 0.12^b^	61.82	3.17 ± 0.12^b^	61.82
Extract (50 mg/kg)	1.34 ± 0.04	1.37 ± 0.08^a^	2.18	1.61 ± 0.05^a^	16.77
Extract (100 mg/kg)	1.45 ± 0.09	2.63 ± 0.17^b^	44.86	2.68 ± 0.17^b^	45.89
Extract (200 mg/kg)	1.16 ± 0.82	2.38 ± 0.14^b^	51.26	2.58 ± 0.11^b^	55.03

Values are mean ± SEM (*n* = 6). Differences in groups were analyzed by one-way analysis of variances (one-way ANOVA) followed by Bonferroni posttest. ^a^ *P* < 0.05 and ^b^ *P* < 0.001 represented highly significant compared to control.

**Table 6:** Effect of methanol extract of *Centella asiatica* leaf on carrageenan-induced rat paw edema.

Treatment	Edema rate (mean ± SE) (*n* = 6)			
	1 h	2 h	3 h	4 h
Control	34.63 ± 1.18	94.90 ± 3.05	122.26 ± 2.24	125.65 ± 2.21
Indomethacin (10 mg/kg)	13.31 ± 0.80^b^ (62.50%)	36.09 ± 2.44^b^ (61.97%)	42.84 ± 2.60^b^ (64.97%)	45.90 ± 2.15^b^ (63.46%)
Extract (100 mg/kg)	30.44 ± 0.63 (12.09%)	69.86 ± 1.62^b^ (26.38%)	102.80 ± 2.26^b^ (16.35%)	111.74 ± 0.90 (11.10%)
Extract (200 mg/kg)	26.26 ± 0.51 (24.16%)	49.29 ± 1.6^b^ (48.06%)	71.29 ± 0.93^b^ (41.68%)	81.23 ± 1.23^b^ (35.35%)

Values are mean ± SEM (*n* = 6). The figure in parenthesis indicates the percentage inhibition. Differences in groups were analyzed by Student's *t*-test compared with control ^b^ *P* < 0.001.
